# Early Postoperative Inflammatory Indices Predict Long‐Term Neuro‐ICU Stay in Patients Requiring Prolonged Care After Supratentorial Brain Tumor Resection: Development and Validation of an Integrated Model

**DOI:** 10.1002/cns.71100

**Published:** 2026-08-22

**Authors:** Biwu Wu, Lichao Wei, Haoyue Yuan, Yu Guo, Jin Hu, Gang Wu

**Affiliations:** ^1^ Department of Neurosurgical Intensive Care Unit, Huashan Hospital Fudan University Shanghai China; ^2^ Department of Neurosurgery, Huashan Hospital Fudan University Shanghai China; ^3^ National Center for Neurological Disorders Shanghai China

**Keywords:** inflammatory indices, length of stay, machine learning, neurosurgical intensive care, prediction model, supratentorial brain tumor

## Abstract

**Background:**

Long‐term stay (LTS) in the neurosurgical intensive care unit (Neuro‐ICU) after supratentorial brain tumor resection disproportionately consumes healthcare resources, but early risk stratification tools remain lacking. We evaluated five early postoperative inflammatory indices (neutrophil‐to‐lymphocyte ratio [NLR], platelet‐to‐lymphocyte ratio [PLR], lymphocyte‐to‐monocyte ratio [LMR], systemic immune‐inflammation index [SII], and systemic inflammation response index [SIRI]) to develop an integrated prediction model.

**Methods:**

This retrospective study included patients requiring > 48‐h Neuro‐ICU care after elective supratentorial tumor resection. Patients were temporally partitioned into derivation (*n* = 432) and validation (*n* = 146) cohorts. Inflammatory indices were measured within 24 h postoperatively. Following least absolute shrinkage and selection operator (LASSO) feature selection, five machine learning classifiers were compared. Model performance was assessed for discrimination, calibration, and clinical utility, alongside subgroup analyses and a series of sensitivity analyses. A nomogram and web‐based risk calculator were developed.

**Results:**

LTS occurred in 47.5% and 49.3% of the derivation and validation cohorts, respectively. LTS patients showed higher early postoperative SIRI and PLR and lower LMR, whereas preoperative indices did not differ. All three were independent predictors of LTS. The integrated model combined these indices with preoperative Karnofsky Performance Status, postoperative steroid use, and retained endotracheal intubation at blood sampling. It yielded an AUC of 0.845 in the derivation cohort and 0.816 in the validation cohort, compared with 0.715 for the clinical baseline model (*p* < 0.001; IDI 0.212; cNRI 0.981). These indices maintained independent predictive value, and model performance remained stable across subgroup and sensitivity analyses.

**Conclusion:**

Early postoperative SIRI, PLR, and LMR independently predict LTS in patients requiring prolonged care (> 48 h) after supratentorial brain tumor resection. An integrated model with these indices and early clinical variables provides reliable risk stratification within 24 postoperative hours.

## Graphical abstract

Early postoperative inflammatory indices (SIRI, PLR, LMR) independently predict long‐term Neuro‐ICU stay after supratentorial brain tumor resection. By combining these biomarkers with clinical variables, the integrated prediction model enables reliable risk stratification and early clinical triage.

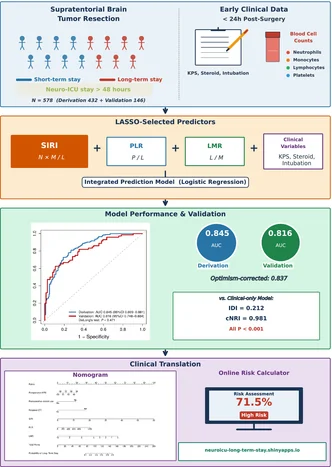

**Abbreviations**
AIC: akaike information criterion
AUC: area under the receiver operating characteristic curve
BBB: blood–brain barrier
CARS: compensatory anti‐inflammatory response syndrome
CI: confidence interval
cNRI: continuous net reclassification improvement
DAMPs: damage‐associated molecular patterns
DCA: decision curve analysis
ERAS: enhanced recovery after surgery
ETI: endotracheal intubation
GBM: gradient boosting machine
GCS: Glasgow Coma Scale
IDI: integrated discrimination improvement
IQR: interquartile range
KNN: k‐nearest neighbors
KPS: Karnofsky Performance Status
LASSO: least absolute shrinkage and selection operator
LMR: lymphocyte‐to‐monocyte ratio
LOS: length of stay
LR: logistic regression
LTS: long‐term stay
Neuro‐ICU: neurosurgical intensive care unit
NLR: neutrophil‐to‐lymphocyte ratio
OR: odds ratio
PLR: platelet‐to‐lymphocyte ratio
PSM: propensity score matching
RF: random forest
SII: systemic immune‐inflammation index
SIRI: systemic inflammation response index
SpO_2_
: peripheral oxygen saturation
STS: short‐term stay
SVM: support vector machine
VIF: variance inflation factor

## 1 Introduction

Globally, neurocritical care has evolved rapidly in recent years, yet it remains burdened by severe resource scarcity [1]. Neurosurgical intensive care units (Neuro‐ICUs) are essential for perioperative management following elective resection of supratentorial brain tumors (the most prevalent adult intracranial neoplasms) [2, 3]. Although most patients require only 1–2 days of routine postoperative observation, stays exceeding 48 h are classified by contemporary practice and enhanced recovery after surgery (ERAS) protocols as non‐routine, prolonged admissions [4, 5, 6, 7, 8, 9]. This high‐risk subgroup, though constituting only 2%–11% of patients, is at risk of progressing to a long‐term stay (LTS) [5, 10, 11]. These LTS patients disproportionately consume Neuro‐ICU resources and increase healthcare expenditures [12, 13]. Therefore, early risk stratification for LTS is crucial for optimizing resource allocation.

Yet, current predictive strategies rely predominantly on static preoperative or intraoperative variables, failing to account for the divergent recovery trajectories observed among patients with similar baseline conditions and surgical trauma [10, 11, 14]. Moreover, prior research largely focuses on predicting brief observation extensions (e.g., > 24 or 48 h), without distinguishing patients who require moderate recovery from those progressing to a true LTS (e.g., ≥ 7 days) [12, 14]. This clinical heterogeneity may reflect the profound systemic inflammatory response provoked by extensive craniotomy [15, 16]. Emerging evidence suggests a potential “crosstalk” wherein circulating inflammatory mediators compromise blood–brain barrier integrity, potentially exacerbating vasogenic cerebral edema and delaying neurologic recovery [17]. Thus, early postoperative blood‐derived inflammatory biomarkers may capture this systemic dysregulation, offering a dynamic and biologically plausible basis for early LTS risk stratification.

Among peripheral blood indices, the neutrophil‐to‐lymphocyte ratio (NLR), platelet‐to‐lymphocyte ratio (PLR), lymphocyte‐to‐monocyte ratio (LMR), and systemic immune‐inflammation index (SII) have demonstrated significant prognostic value in neuro‐oncology [18, 19, 20, 21]. More recently, the systemic inflammation response index (SIRI), which integrates peripheral neutrophil, monocyte, and lymphocyte counts, has shown favorable predictive accuracy for postoperative complications and outcomes in various brain tumors compared with conventional markers like NLR [22, 23, 24]. Importantly, while the utility of these inflammatory signatures has been established for predicting length of ICU stay and hospital resource utilization across various clinical conditions [25, 26, 27, 28], their comparative efficacy and collective utility for forecasting acute Neuro‐ICU trajectories, specifically LTS, remain largely unexplored.

To address this gap, this study aimed to evaluate and compare early postoperative (< 24 h) inflammatory indices (NLR, PLR, LMR, SII, SIRI) for predicting long‐term Neuro‐ICU stay (≥ 7 days) in patients requiring prolonged care (> 48 h) following elective supratentorial brain tumor resection. We sought to develop and temporally validate an integrated early prediction model combining the optimal inflammatory markers with clinical variables to provide a practical clinical tool for early risk stratification and resource allocation.

## 2 Methods

### 2.1 Study Design and Population

This single‐center retrospective study was conducted at the 94‐bed Neuro‐ICU of Huashan Hospital, Fudan University, between January 1, 2023, and December 31, 2024. The protocol was approved by the Institutional Review Board of Huashan Hospital (No. 2025‐648), with informed consent waived due to the retrospective design, and complied with the Declaration of Helsinki. The study was registered with the Chinese Clinical Trial Registry (No. ChiCTR2500104426) and reported following the STROBE and TRIPOD+AI guidelines [29, 30] (completed checklists provided as Supporting Information).

Consecutive patients admitted to the Neuro‐ICU were screened. Inclusion criteria were: (1) Neuro‐ICU stay > 48 h; and (2) radiologically and histopathologically confirmed supratentorial tumor. Exclusion criteria were: (1) age < 18 years; (2) preoperative infection (pulmonary, intracranial, or urinary tract); (3) preoperative use of corticosteroids, immunosuppressants, radiotherapy, or chemotherapy; (4) history of hematologic disorders; and (5) missing essential baseline or laboratory data. Patients with subsequent Neuro‐ICU readmissions were excluded to avoid ambiguity in length of stay (LOS). Eligible patients enrolled between January 2023 and June 2024 formed the derivation cohort, and those between July and December 2024 formed the temporal validation cohort.

### 2.2 Data Collection and Variable Definitions

Two investigators (H.Y. and Y.G.), blinded to the study hypothesis, retrospectively extracted data from the electronic medical record system, including demographics, medical history, and preoperative, intraoperative, and postoperative variables. Among 608 eligible patients, 30 (4.9%) had missing data and were excluded. All analyses used complete cases without imputation.

Preoperative Karnofsky Performance Status (KPS) was recorded as a continuous variable (0–100). Tumor type was dichotomized as malignant (high‐grade glioma, metastasis) versus non‐malignant, and tumor site as deep (skull base, basal ganglia, ventricle, or pineal region) versus lobar. The admission Glasgow Coma Scale (GCS) score, blood pressure, heart rate, body temperature, and peripheral oxygen saturation (SpO_2_) were recorded immediately upon Neuro‐ICU arrival; for patients not fully awake from anesthesia, standardized pain stimulation was applied to assess best responses, and the verbal component was systematically scored as 1 for intubated patients. The first postoperative blood samples were drawn the following morning (5–7 a.m.), with the exact sampling time recorded. Retained endotracheal intubation (ETI) at blood sampling was defined as the patient remaining intubated at the time of this blood draw. Postoperative steroid use was defined as a single dose (5 mg dexamethasone or 40 mg methylprednisolone) administered between surgery completion and the morning blood draw. Late postoperative variables (≤ 48 h) included prolonged ETI (failure to extubate), seizures (clinically or electrographically observed), and pulmonary infection (diagnosed clinically and confirmed by chest computed tomography). Postoperative hemorrhage and unplanned reoperation (≤ 48 h) were defined according to established criteria [31, 32].

Five inflammatory indices were calculated from complete blood counts obtained both preoperatively and early postoperatively: SIRI = (neutrophil × monocyte)/lymphocyte; NLR = neutrophil/lymphocyte; PLR = platelet/lymphocyte; LMR = lymphocyte/monocyte; and SII = (platelet × neutrophil)/lymphocyte.

### 2.3 Outcome Definition

The primary outcome was LTS, defined as Neuro‐ICU LOS ≥ 7 days, with LOS < 7 days classified as short‐term stay (STS). No standardized definition of LTS exists after intracranial tumor resection. We therefore set the threshold immediately above the cohort median of 6.0 days, which yielded two groups of comparable size. Prolonged Neuro‐ICU stays were primarily attributable to interventions unavailable on general wards, including advanced life support, invasive neuromonitoring, and intensive complication management.

### 2.4 Statistical Analyses

#### 2.4.1 Descriptive Analyses and Feature Selection

Continuous variables were expressed as mean ± SD or median (IQR) and compared using the *t*‐test or Wilcoxon rank‐sum test. Categorical variables were presented as frequencies (percentages) and compared using the chi‐square or Fisher's exact test. Spearman correlation analysis and a correlation heatmap were used to explore inter‐variable associations prior to feature selection. Least absolute shrinkage and selection operator (LASSO) regression with 10‐fold cross‐validation was employed for feature selection, using a candidate pool of 19 variables drawn from those with *p* < 0.05 in univariable analysis (205 LTS events; events‐per‐variable = 10.8).

#### 2.4.2 Algorithm Selection and Model Construction

The selected features were evaluated across five machine learning classifiers: logistic regression (LR), random forest (RF), gradient boosting machine (GBM), support vector machine (SVM), and k‐nearest neighbors (KNN). Discrimination was assessed by the area under the receiver operating characteristic curve (AUC) and compared using DeLong's test; calibration was evaluated using calibration plots; and clinical utility was evaluated using decision curve analysis (DCA). Based on initial comparative performance, LR was selected to construct three multivariable models: an early clinical base model (Model 1); an integrated early prediction model (Model 2) incorporating the selected clinical variables and inflammatory indices; and a late‐integrated model (Model 3) further adjusting for late postoperative complications. Multicollinearity was assessed via the variance inflation factor (VIF). Optimal cutoffs were determined by maximizing the Youden index. Incremental predictive value was quantified by the integrated discrimination improvement (IDI), continuous net reclassification improvement (cNRI), and Akaike information criterion (AIC). Relative feature importance was ranked using the Wald chi‐square statistic.

#### 2.4.3 Model Validation and Sensitivity Analyses

Model performance was internally validated using 1000 bootstrap resamples within the derivation cohort, followed by external validation in the temporal cohort. In both cohorts, discrimination was assessed by AUC, calibration was evaluated using calibration plots together with the Hosmer‐Lemeshow test, Brier score, and calibration slope and intercept, and clinical utility was assessed using DCA. Subgroup analyses with interaction tests assessed the consistency of SIRI and the discriminative stability of Model 2 across prespecified clinical strata.

Several sensitivity analyses were conducted. First, patients were stratified into high‐ and low‐SIRI groups based on the optimal cutoff, and 1:1 propensity score matching (PSM; caliper width = 0.20) was applied, followed by conditional logistic regression. Second, the outcome definition was varied across LTS thresholds of 4–10 days, with the coefficients of the primary model held fixed. Third, the cohort was re‐partitioned by calendar year and the analysis repeated. Fourth, the specification of the airway variable was varied, and adjustment was made for time to blood sampling and other perioperative factors. Fifth, the early postoperative inflammatory indices were adjusted for their preoperative values. Sixth, additional early clinical variables and a score combining comorbidities and preoperative functional status were added to the model.

To facilitate clinical translation, Model 2 was visualized as a nomogram and developed into a web‐based risk calculator using the R‐Shiny framework. All analyses were performed using R software (version 4.4.2). A two‐sided *p* < 0.05 was considered statistically significant.

## 3 Results

**FIGURE 1:**
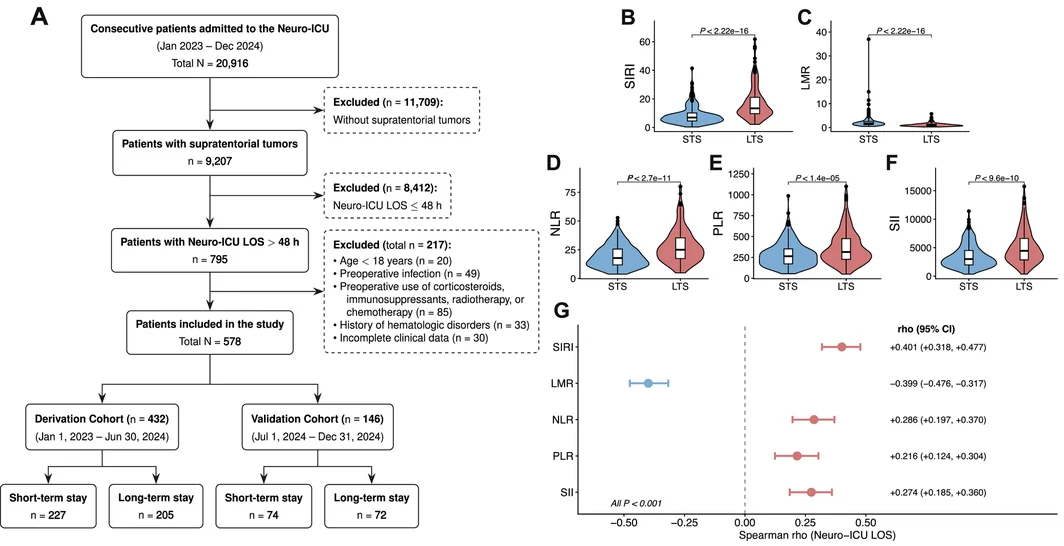
Patient enrollment and early postoperative inflammatory profiles. (A) Flowchart of patient selection. (B–F) Violin plots comparing early postoperative SIRI (B), LMR (C), NLR (D), PLR (E), and SII (F) between the short‐term stay and long‐term stay groups in the derivation cohort. (G) Forest plot of the Spearman correlation coefficients (*ρ*) between each inflammatory index and Neuro‐ICU LOS. CI, confidence interval; LMR, lymphocyte‐to‐monocyte ratio; LOS, length of stay; LTS, long‐term stay; Neuro‐ICU, neurosurgical intensive care unit; NLR, neutrophil‐to‐lymphocyte ratio; PLR, platelet‐to‐lymphocyte ratio; SII, systemic immune‐inflammation index; SIRI, systemic inflammation response index; STS, short‐term stay.

A total of 20,916 consecutive postoperative Neuro‐ICU admissions were screened, of which 9207 followed resection of a supratentorial tumor. Among these, 795 patients (8.6%) remained in the Neuro‐ICU for more than 48 h. After applying the exclusion criteria, 217 patients were removed, including 30 with incomplete baseline or laboratory data. The remaining 578 patients were temporally divided into a derivation cohort (*n* = 432; January 2023–June 2024) and a validation cohort (*n* = 146; July 2024–December 2024) (Figure 1A).

### 3.1 Baseline Characteristics

In the derivation cohort, the median age was 60.0 years (IQR 48.0–67.0), 217 (50.2%) were female, and 205 (47.5%) experienced LTS. The validation cohort comprised 65 (44.5%) females with a median age of 61.0 years (IQR 53.0–69.0) and an LTS rate of 49.3% (72/146). Baseline characteristics were well balanced between the two cohorts, except for hypertension, lymphocyte count, alanine aminotransferase, and Neuro‐ICU LOS (Table 1). Comparisons between STS and LTS groups within the derivation cohort showed that LTS patients had significantly lower preoperative KPS and Neuro‐ICU admission GCS scores, longer operative times, higher rates of retained ETI at blood sampling, and a different distribution of tumor type and tumor site (all *p* < 0.05; Table S1). Although preoperative inflammatory indices did not differ between the two groups (Table S2), LTS patients exhibited significantly higher early postoperative levels of SIRI, NLR, PLR, and SII, alongside lower LMR (all *p* < 0.001; Figure 1B–F). Spearman correlation analysis showed that SIRI and LMR had the strongest correlations with Neuro‐ICU LOS (*ρ* = 0.401, 95% CI 0.318–0.477, and *ρ* = −0.399, 95% CI −0.476 to −0.317, respectively), followed by NLR (*ρ* = 0.286), SII (*ρ* = 0.274), and PLR (*ρ* = 0.216), all *p* < 0.001 (Figure 1G).

**TABLE 1:** Baseline characteristics of patients in the derivation and validation cohorts.

Variables	Derivation cohort (*N* = 432)	Validation cohort (*N* = 146)	*p*
Age, years	60.0 (48.0, 67.0)	61.0 (53.0, 69.0)	0.065
Female	217 (50.2%)	65 (44.5%)	0.272
Hypertension	206 (47.7%)	88 (60.3%)	0.011
Diabetes	112 (25.9%)	49 (33.6%)	0.094
Preoperative KPS	90.0 (80.0, 90.0)	90.0 (80.0, 90.0)	0.876
Tumor type			0.561
High‐grade glioma	208 (48.1%)	77 (52.7%)	
Meningioma	107 (24.8%)	36 (24.7%)	
Pituitary adenoma	37 (8.6%)	14 (9.6%)	
Metastasis	30 (6.9%)	10 (6.8%)	
Low‐grade glioma	27 (6.2%)	4 (2.7%)	
Other	23 (5.3%)	5 (3.4%)	
Tumor site			0.302
Lobe	271 (62.7%)	106 (72.6%)	
Skull base	91 (21.1%)	22 (15.1%)	
Basal ganglia	44 (10.2%)	13 (8.9%)	
Ventricle	20 (4.6%)	4 (2.7%)	
Pineal region	6 (1.4%)	1 (0.7%)	
Tumor diameter, cm	5.0 (3.6, 6.0)	5.0 (4.2, 6.0)	0.259
Tumor status			0.841
Initial onset	380 (88%)	130 (89%)	
Recurrence	52 (12%)	16 (11%)	
Surgical approach			0.145
Craniotomy	350 (81%)	127 (87%)	
Endoscopic surgery	47 (10.9%)	8 (5.5%)	
Keyhole surgery	35 (8.1%)	11 (7.5%)	
Operative time, h	5.0 (4.0, 7.0)	5.0 (4.0, 6.0)	0.189
Intraoperative blood loss, mL	400.0 (300.0, 500.0)	400.0 (300.0, 600.0)	0.063
Intraoperative RBC transfusion			0.837
≤ 400 mL	386 (89.4%)	132 (90.4%)	
> 400 mL	46 (10.6%)	14 (9.6%)	
Postoperative steroid use	320 (74.1%)	105 (71.9%)	0.688
Neuro‐ICU admission GCS score^a^	4.0 (3.0, 10.0)	4.0 (3.0, 8.0)	0.884
Postoperative SBP, mmHg	139.0 (126.0, 150.0)	137.5 (125.0, 146.0)	0.128
Postoperative DBP, mmHg	82.0 (74.0, 90.0)	81.5 (72.0, 89.0)	0.500
Postoperative SpO_2_, %	99.0 (98.0, 100.0)	99.0 (98.0, 100.0)	0.408
Postoperative heart rate	88.0 (77.0, 100.0)	86.0 (77.0, 98.0)	0.502
Postoperative body temperature, °C	36.7 (36.5, 37.0)	36.7 (36.5, 37.0)	0.926
Time to blood sampling, h	11.0 (8.0, 13.5)	11.5 (9.0, 13.5)	0.518
Retained ETI at blood sampling	269 (62.3%)	96 (65.8%)	0.512
White blood cells, × 10^9^/L	14.0 (11.7, 17.2)	14.6 (12.0, 17.9)	0.102
Neutrophil count, × 10^9^/L	12.7 (10.6, 15.7)	13.5 (10.8, 16.3)	0.085
Lymphocyte count, × 10^9^/L	0.6 (0.4, 0.9)	0.7 (0.5, 0.9)	0.037
Monocyte count, × 10^9^/L	0.5 (0.3, 0.8)	0.6 (0.3, 0.8)	0.408
Red blood cells, × 10^12^/L	4.0 (3.7, 4.4)	4.1 (3.7, 4.4)	0.338
Hemoglobin, g/L	120.0 (111.0, 132.0)	121.0 (111.0, 132.0)	0.719
Platelet, × 10^9^/L	181.0 (138.5, 214.0)	178.0 (146.0, 222.0)	0.649
Alanine aminotransferase, U/L	19.0 (14.0, 30.0)	27.0 (17.0, 36.0)	< 0.001
Creatinine, μmol/L	63.0 (53.0, 74.0)	65.0 (54.0, 76.0)	0.396
Systemic inflammation response index	9.6 (5.8, 16.5)	10.9 (5.8, 17.4)	0.478
Neutrophil‐to‐lymphocyte ratio	21.2 (14.0, 29.8)	18.9 (14.2, 26.5)	0.267
Platelet‐to‐lymphocyte ratio	293.2 (197.2, 421.2)	256.4 (188.9, 354.2)	0.068
Lymphocyte‐to‐Monocyte Ratio	1.3 (0.8, 2.0)	1.2 (0.9, 2.0)	0.957
Systemic Immune‐Inflammation Index	3502.5 (2353.9, 5539.2)	3569.1 (2570.2, 4836.6)	0.618
Prothrombin time, s	11.3 (10.8, 11.7)	11.2 (10.8, 11.6)	0.621
Activated partial thromboplastin time, s	26.8 (25.2, 28.2)	26.4 (25.3, 27.8)	0.261
Prolonged ETI (at 48 h)	108 (25%)	43 (29.5%)	0.342
Seizures (≤ 48 h)	41 (9.5%)	8 (5.5%)	0.183
Pulmonary infection (≤ 48 h)	209 (48.4%)	75 (51.4%)	0.597
Postoperative hemorrhage (≤ 48 h)	127 (29.4%)	48 (32.9%)	0.492
Unplanned reoperation (≤ 48 h)	38 (8.8%)	10 (6.8%)	0.573
Long‐term NICU stay (≥ 7 days)	205 (47.5%)	72 (49.3%)	0.769
Neuro‐ICU length of stay, days	6.0 (4.0, 9.0)	6.0 (5.0, 10.0)	0.043

*Note:* Data are presented as *n* (%) or median (IQR).

Abbreviations: DBP, diastolic blood pressure; ETI, endotracheal intubation; GCS, Glasgow Coma Scale; KPS, Karnofsky Performance Status; Neuro‐ICU, neurosurgical intensive care unit; RBC, red blood cells; SBP, systolic blood pressure; SpO_2_, peripheral oxygen saturation.

^a^ 80.6% (466/578) of patients were intubated and/or sedated upon Neuro‐ICU arrival, accounting for the low median GCS score.

### 3.2 Feature Selection and Algorithm Optimization

**FIGURE 2:**
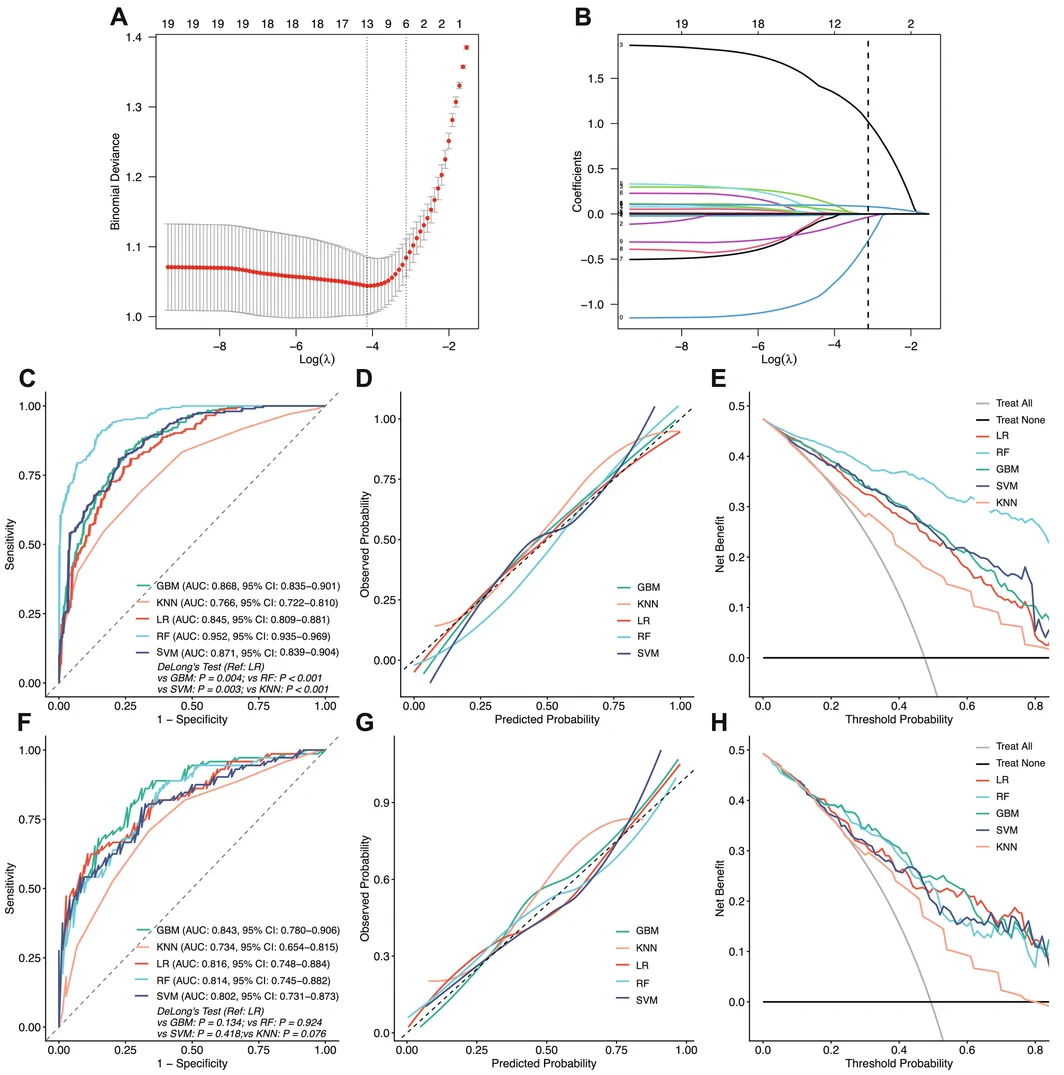
Feature selection and comparison of five machine learning classifiers. (A) Cross‐validation curve from LASSO regression, showing the binomial deviance against log(*λ*). (B) Coefficient profiles of candidate variables across the LASSO regularization path. (C–E) Performance of five machine learning classifiers in the derivation cohort, including ROC curves (C), calibration curves (D), and decision curves (E). (F–H) Corresponding performance in the temporal validation cohort, including ROC curves (F), calibration curves (G), and decision curves (H). AUC, area under the receiver operating characteristic curve; CI, confidence interval; GBM, gradient boosting machine; KNN, k‐nearest neighbors; LASSO, least absolute shrinkage and selection operator; LR, logistic regression; RF, random forest; ROC, receiver operating characteristic; SVM, support vector machine.

Given the high multicollinearity among the initial features (Figure S1), LASSO regression was applied to the 19 candidate variables. Six predictors retained non‐zero coefficients: preoperative KPS, postoperative steroid use, retained ETI, SIRI, PLR, and LMR (Figure 2A,B). These features were subsequently evaluated across five machine learning classifiers (LR, RF, GBM, SVM, and KNN). RF achieved the highest derivation AUC (0.952), but its performance fell markedly in the validation cohort (0.814). GBM performed similarly to LR in the validation cohort (0.843 vs. 0.816, *p* = 0.134) (Figure 2C,F). The LR model maintained cross‐cohort stability (derivation AUC: 0.845; validation AUC: 0.816), with well‐calibrated predictions (Figure 2D,G) and favorable net clinical benefit (Figure 2E,H). Given its stable discrimination and inherent interpretability, LR was selected as the foundational algorithm.

### 3.3 Development and Validation of the Integrated Model

After confirming the absence of multicollinearity (all VIF < 2.0; Table S3), multivariable logistic regression identified three independent clinical predictors: preoperative KPS (OR 0.98, 95% CI 0.97–1.00; *p* = 0.030), postoperative steroid use (OR 0.33, 95% CI 0.18–0.58; *p* < 0.001), and retained ETI at blood sampling (OR 5.72, 95% CI 3.37–10.00; *p* < 0.001) to form the clinical baseline model (Model 1). By integrating three independent inflammatory indices: SIRI (OR 1.11, 95% CI 1.06–1.16; *p* < 0.001), PLR (OR 1.19 per 100‐unit increase, 95% CI 1.03–1.37; *p* = 0.020), and LMR (OR 0.68, 95% CI 0.45–0.96; *p* = 0.050), we developed the final integrated model (Model 2; Table 2).

**TABLE 2:** Multivariable logistic regression models for predicting long‐term stay.

Clinical variables	Model 1		Model 2		Model 3	
	OR (95% CI)	*p*	OR (95% CI)	*p*	OR (95% CI)	*p*
Preoperative KPS	0.98 (0.97–1.00)	0.012	0.98 (0.97–1.00)	0.030	0.98 (0.96–0.99)	0.006
Postoperative steroid use	0.43 (0.26–0.70)	< 0.001	0.33 (0.18–0.58)	< 0.001	0.32 (0.16–0.62)	< 0.001
Retained ETI at blood sampling	4.56 (2.93–7.22)	< 0.001	5.72 (3.37–10.00)	< 0.001	2.31 (1.22–4.42)	0.011
SIRI	—	—	1.11 (1.06–1.16)	< 0.001	1.08 (1.03–1.13)	0.002
PLR (per 100‐unit increase)	—	—	1.19 (1.03–1.37)	0.020	1.23 (1.04–1.46)	0.020
LMR	—	—	0.68 (0.45–0.96)	0.050	0.63 (0.41–0.92)	0.028
Prolonged ETI (at 48 h)	—	—	—	—	3.90 (1.81–8.76)	< 0.001
Pulmonary infection (≤ 48 h)	—	—	—	—	4.16 (2.32–7.55)	< 0.001
Postoperative hemorrhage (≤ 48 h)	—	—	—	—	2.15 (1.10–4.26)	0.026
Unplanned reoperation (≤ 48 h)	—	—	—	—	1.03 (0.34–3.35)	0.954

*Note:* “—”, variable not included in the model. Model 1: Preoperative KPS, postoperative steroid use, and retained endotracheal intubation at blood sampling. Model 2: Model 1 + SIRI + PLR + LMR. Model 3: Model 2 + late postoperative complications.

Abbreviations: CI, confidence interval; ETI, endotracheal intubation; KPS, Karnofsky Performance Status; LMR, lymphocyte‐to‐monocyte ratio; OR, odds ratio; PLR, platelet‐to‐lymphocyte ratio; SIRI, systemic inflammation response index.

**FIGURE 3:**
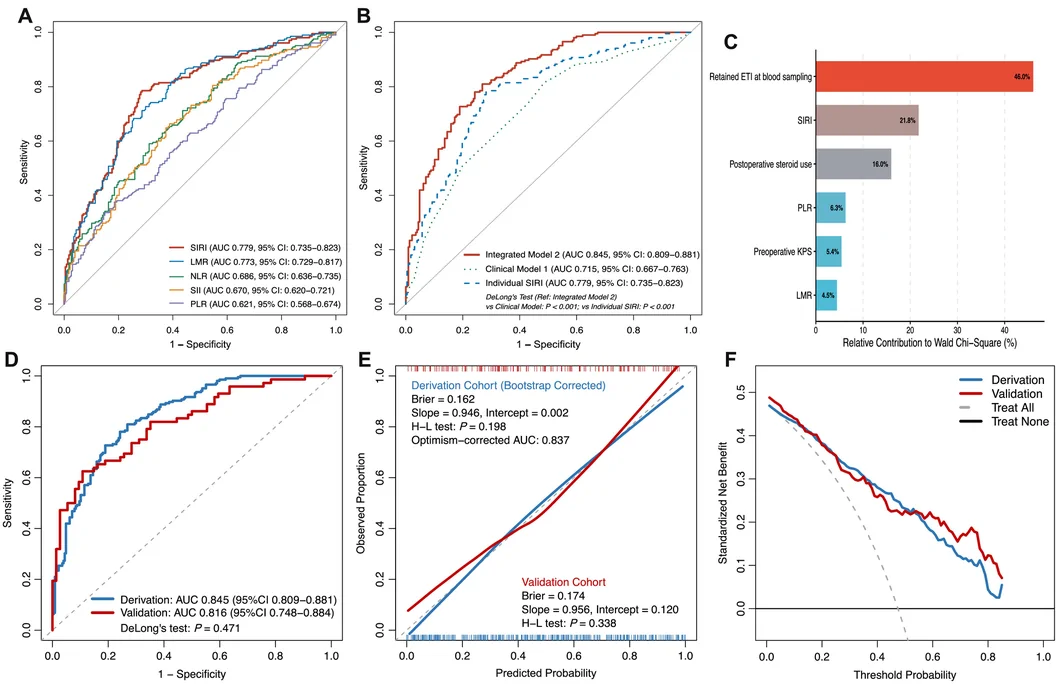
Evaluation and validation of the integrated prediction model. (A) ROC curves of the five individual inflammatory indices in the derivation cohort. (B) ROC curves of the integrated Model 2, the clinical‐only Model 1, and individual SIRI in the derivation cohort, with between‐model comparisons by DeLong's test. (C) Relative contributions of the six retained predictors to the model, ranked by the Wald chi‐square statistic. (D) ROC curves of Model 2 in the derivation and temporal validation cohorts. (E) Calibration curves of Model 2 in both cohorts. (F) Decision curve analysis of Model 2 in both cohorts. AUC, area under the receiver operating characteristic curve; CI, confidence interval; ETI, endotracheal intubation; H–L, Hosmer–Lemeshow; KPS, Karnofsky Performance Status; LMR, lymphocyte‐to‐monocyte ratio; PLR, platelet‐to‐lymphocyte ratio; ROC, receiver operating characteristic; SIRI, systemic inflammation response index.

SIRI and LMR showed the highest discriminative ability among individual inflammatory indices (AUC 0.779 and 0.773; *p* = 0.565), and both outperformed NLR, SII, and PLR (all *p* ≤ 0.001; Figure 3A, Table S4). Model 2 significantly outperformed both the clinical Model 1 and SIRI alone (AUC 0.845 vs. 0.715 and 0.779; both *p* < 0.001; Figure 3B). Reclassification analysis demonstrated substantial improvements in Model 2 (IDI 0.212, cNRI 0.981; Table S5). At the optimal cutoff of 0.449, Model 2 achieved 81.0% sensitivity and 73.1% specificity (Table S6). Wald chi‐square analysis identified retained ETI (46.0%) and SIRI (21.8%) as the primary predictive drivers (Figure 3C). These early inflammatory indices remained independently predictive even in Model 3 after adjusting for late postoperative complications (Table 2).

Internal validation of Model 2 via 1000 bootstrap resamples yielded an optimism‐corrected AUC of 0.837. The model maintained consistent discrimination in the temporal validation cohort (AUC 0.816, 95% CI 0.748–0.884; *p* = 0.471 vs. derivation; Figure 3D). Calibration was confirmed in both cohorts (derivation: Brier score 0.162, slope 0.946; validation: Brier score 0.174, slope 0.956; Figure 3E). DCA demonstrated net clinical benefit across threshold probabilities of 1.0%–85.0% in both cohorts (Figure 3F).

### 3.4 Subgroup and Sensitivity Analyses

**FIGURE 4:**
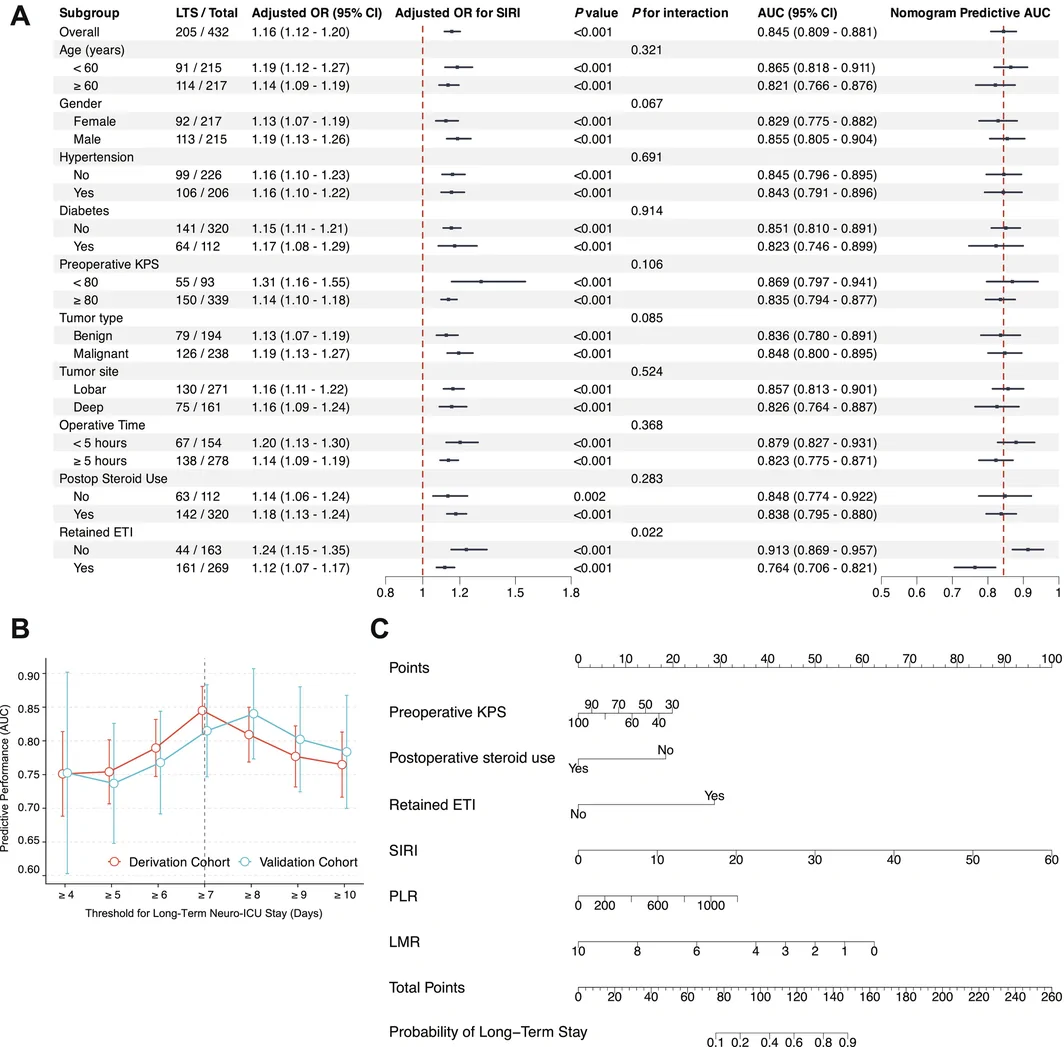
Subgroup analyses, threshold sensitivity, and the clinical nomogram. (A) Forest plot of subgroup analyses showing the adjusted odds ratio (OR) of SIRI for LTS and the predictive performance (AUC) of the integrated model across 10 prespecified clinical strata, with the overall estimate at the top; the red dashed lines mark an OR of 1.0 and the overall AUC of 0.845, respectively. (B) Predictive performance (AUC) of the integrated model across LTS thresholds ranging from 4 to 10 days in the derivation and temporal validation cohorts. (C) Nomogram derived from the integrated model. AUC, area under the receiver operating characteristic curve; ETI, endotracheal intubation; KPS, Karnofsky Performance Status; LMR, lymphocyte‐to‐monocyte ratio; LTS, long‐term stay; Neuro‐ICU, neurosurgical intensive care unit; OR, odds ratio; PLR, platelet‐to‐lymphocyte ratio; SIRI, systemic inflammation response index.

Subgroup analyses demonstrated that the association between SIRI and LTS risk remained robust across all prespecified clinical strata (*p* for interaction > 0.05), with the exception of retained ETI (*p* for interaction = 0.022), where SIRI remained predictive in both subgroups (both *p* < 0.001). Model discrimination was higher in patients without retained ETI at blood sampling (AUC 0.913) than in those with retained ETI (AUC 0.764), and remained stable across the other strata (Figure 4A).

Sensitivity analyses further confirmed the robustness of our findings. After 1:1 PSM (118 well‐balanced pairs; Table S7, Figure S2), SIRI remained independently predictive both continuously (OR 1.24, 95% CI 1.11–1.38; *p* < 0.001) and categorically (SIRI ≥ 9.26 vs. < 9.26: OR 9.60, 95% CI 3.82–24.11; *p* < 0.001; Table S8). Dynamic evaluation across multiple LTS definitions (4–10 days) showed that the model's performance peaked at the 7‐day threshold in the derivation cohort and remained stable in the validation cohort (Figure 4B, Table S8). Re‐partitioning the cohort by calendar year yielded comparable discrimination (derivation AUC 0.833, validation AUC 0.835; Table S9).

Model performance was robust to alternative specifications of the airway variable (Table S10). Removing retained ETI reduced discrimination, but the model remained acceptable in both cohorts (derivation AUC 0.803, validation AUC 0.802), with SIRI, PLR, and LMR all remaining independent predictors. Preoperative inflammatory indices discriminated poorly for LTS (AUC 0.538–0.565), and early postoperative SIRI and PLR remained independently associated with LTS after adjustment for their preoperative values (Table S11). Adding Neuro‐ICU admission GCS, deep tumor location, postoperative SpO_2_, systolic blood pressure, and age did not improve discrimination (AUC 0.849 vs. 0.845; *p* = 0.227; Table S12). A composite index of hypertension, diabetes, and reduced preoperative KPS (< 70) was associated with LTS in univariable analysis but not after adjustment (Table S13).

### 3.5 Clinical Nomogram and Web‐Based Calculator

To facilitate clinical application, Model 2 was converted into a predictive nomogram (Figure 4C). An interactive web‐based risk calculator was also developed (https://neuroicu‐long‐term‐stay.shinyapps.io/NICU_Calculator/), enabling clinicians to generate individualized LTS probabilities.

## 4 Discussion

In this study, we demonstrated that early postoperative SIRI, PLR, and LMR emerged as independent predictors of long‐term Neuro‐ICU stay (LTS) in patients requiring prolonged care (> 48 h) following elective supratentorial brain tumor resection. Through a comparative analysis of five machine learning classifiers, logistic regression was selected to construct our integrated model (Model 2) due to its optimal cross‐cohort stability and avoidance of overfitting. Incorporating these inflammatory indices alongside core clinical variables, Model 2 achieved superior discrimination across both the derivation (optimism‐corrected AUC 0.837) and temporal validation cohorts (AUC 0.816). The model exhibited excellent calibration and provided substantial incremental predictive value over clinical features alone (IDI 0.212; cNRI 0.981). Supported by its confirmed net clinical benefit, we translated Model 2 into a dynamic web‐based calculator that facilitates early, individualized risk stratification at the bedside.

In our cohort, patients requiring prolonged care (> 48 h) accounted for 8.6% (795/9207) of all screened supratentorial tumor resections, and nearly half (47.9%, 277/578) of the final analytic cohort experienced LTS. Previous studies have shown that patients with an ICU stay exceeding 14 days constituted merely 6.6% of admissions but occupied > 40% of all ICU bed‐days [12]. Daily Neuro‐ICU costs following supratentorial brain tumor resection have been reported to range from $1504 to $2376 [13, 33]. Currently, no standardized definition for “prolonged stay” exists, with durations ranging from 1 to 10 days across centers and studies [34, 35, 36, 37, 38, 39]. We defined prolonged stay as > 48 h to exclude the routine postoperative observation window, and set the LTS threshold at the first full day beyond the cohort median, which balanced the two groups for stable model estimation. Acknowledging the wide variation in definitions, we performed a dynamic threshold analysis across LTS definitions from 4 to 10 days, and the model maintained stable discrimination in both the derivation and validation cohorts (AUC 0.73–0.85; Table S8), showing consistent performance across a range of clinically plausible thresholds.

Despite this substantial burden, existing predictive strategies have significant limitations. Most studies on elective supratentorial tumor resection focused narrowly on whether routine postoperative Neuro‐ICU admission is necessary, rather than identifying patients who, once admitted, will progress to a true LTS [7, 8, 9]. The few existing models predominantly rely on static preoperative variables (e.g., age, KPS, tumor type) or delayed complications (e.g., pulmonary infection, hemorrhage) [10, 11, 36], which either fail to capture the dynamic inflammatory response to surgical trauma or manifest too late for early intervention. Previous studies mainly focused on preoperative inflammatory indices [22, 23, 24, 40], although evidence shows that surgery‐induced immune activation occurs within 24 h and is closely linked to subsequent complications [15, 16]. Our study is, to our knowledge, the first to specifically target the LTS subgroup by developing and temporally validating an integrated early prediction model incorporating early postoperative blood‐derived inflammatory markers.

The predictive value of these indices may be explained, at least in part, by the early postoperative immune‐inflammatory response and the subsequent crosstalk between the peripheral circulation and the central nervous system [17]. Surgical trauma triggers sterile systemic inflammation primarily via damage‐associated molecular patterns (DAMPs), activating Toll‐like receptors on cerebral endothelial cells and driving neutrophil recruitment, monocyte infiltration, and lymphocyte suppression [16, 41, 42, 43]. The blood–brain barrier (BBB) acts not merely as a passive filter but as a dynamic biosensor that communicates this peripheral inflammatory storm to the brain [17, 44]. The SIRI formula directly quantifies this triad as pro‐inflammatory drivers (neutrophils) multiplied by inflammatory amplifiers (monocytes) divided by immune regulators (lymphocytes). The monocyte component of SIRI reflects a key pathway linking peripheral inflammation to central pathology. Under inflammatory conditions, circulating monocytes can infiltrate the brain via the compromised BBB, differentiate into pro‐inflammatory macrophages, and promote neuro‐inflammation and vasogenic cerebral edema [44, 45, 46]. This peripheral–central crosstalk is further supported by evidence that systemic inflammation can degrade tight junction proteins such as claudin‐5 and occludin, thereby increasing BBB solute permeability even in the absence of overt structural damage [17, 44].

The inclusion of PLR accounts for the thrombo‐inflammatory component of this crosstalk. Surgical trauma activates platelets, which promote microvascular thrombosis and release abundant pro‐inflammatory cytokines [47, 48]; therefore, incorporating PLR into our integrated model captures this thrombo‐inflammatory dimension that SIRI alone cannot. Furthermore, a low LMR may serve as a surrogate for postoperative immunosuppression. Severe surgical stress is associated with a compensatory anti‐inflammatory response syndrome (CARS), characterized by lymphocyte depletion and monocyte dysfunction [15, 16, 49]. This state may increase vulnerability to delayed secondary hits, such as nosocomial infections, which may contribute to prolonged Neuro‐ICU care [15, 16]. Together, elevated SIRI and PLR, along with reduced LMR, offer a multidimensional readout of the peripheral–central interaction spanning neuro‐inflammation, microvascular dysfunction, and immune exhaustion. Such inflammatory crosstalk between the systemic circulation and the central nervous system has likewise been reported in other disease settings [50, 51].

These mechanistic considerations align with our clinical observations. Preoperatively, inflammatory indices were low and comparable between the STS and LTS groups (median SIRI 0.8 vs. 0.9; Table S2). Following surgery, we observed a marked systemic inflammatory response, with the median postoperative SIRI rising to 9.6. LTS patients exhibited significantly higher early postoperative SIRI and PLR, along with lower LMR, compared with STS patients (all *p* < 0.001). Spearman analysis showed that SIRI and LMR had the strongest correlations with Neuro‐ICU LOS, in opposite directions. These observations suggest that the identified inflammatory signatures are dynamic, surgery‐associated indicators of physiological burden rather than preexisting conditions.

To further investigate the association between early postoperative immune status and susceptibility to secondary hits, we utilized PSM to mitigate baseline confounding. In the matched cohort, patients with high early SIRI experienced higher rates of late postoperative complications, including pulmonary infections and postoperative hemorrhage (Table S7). This clinical phenotype is consistent with the hypothesis of CARS‐related vulnerability discussed above. To evaluate whether these markers act as surrogates for these subsequent complications, we constructed a late‐integrated model (Model 3). After adjusting for delayed events, SIRI, PLR, and LMR remained independent predictors of LTS. These findings indicate that early postoperative inflammatory markers provide independent prognostic value for a protracted Neuro‐ICU trajectory, beyond what is explained by overt clinical complications alone.

The choice of LR as the foundational algorithm warrants emphasis. Although RF and GBM achieved higher derivation performance, they overfitted during temporal validation and were less reliable across cohorts, whereas LR maintained stable discrimination and offered the interpretability essential for clinical translation. The model was also robust across subgroup and sensitivity analyses. The association between SIRI and LTS remained consistent across most clinical strata, and although a significant interaction was observed for retained ETI, SIRI predicted LTS in both intubated and non‐intubated patients. We defined intubation status at the time of blood sampling rather than later, even though later timepoints gave a higher AUC (Table S10). A longer intubation naturally lies closer to the outcome and carries more prognostic weight, but this offers limited early warning, when intervention is still possible. Frailty, measured by the modified 5‐item frailty index, has been reported as an independent predictor of prolonged intensive care and hospital stay after brain tumor surgery [36]. In our cohort, a composite index of hypertension, diabetes, and reduced preoperative KPS was associated with LTS on univariable analysis but was no longer significant after adjustment for KPS (Table S13), suggesting that its prognostic signal may overlap with baseline functional status.

To facilitate bedside translation, we converted Model 2 into a nomogram and deployed it as a web‐based calculator. For patients who remain in the Neuro‐ICU beyond 48 h, this tool can be applied using data collected within the first 24 h after surgery. At the optimal cutoff (sensitivity 81.0%, specificity 73.1%), DCA confirmed net clinical benefit across a wide range of risk thresholds. For low‐risk patients, early transfer to general wards could reduce Neuro‐ICU bed occupancy and costs. For high‐risk patients, proactive measures such as intensified monitoring, early intubation management, and appropriate anti‐inflammatory therapy may be considered. This approach supports a shift from empirical to evidence‐based resource allocation in the Neuro‐ICU.

This study has several limitations. First, its single‐center, retrospective nature carries risks of selection and information biases, despite PSM and blinded data extraction. Second, residual confounding from unmeasured factors (e.g., detailed anesthetic protocol, intraoperative hemodynamic fluctuation, tumor molecular subtype) cannot be ruled out. Third, the inflammatory indices were measured at a single early timepoint, and serial sampling might better capture the inflammatory response over time. Fourth, although validated in a temporal cohort, the generalizability of our findings warrants confirmation in prospective, multi‐institutional studies. Fifth, our exclusion criteria (e.g., preoperative infection, immunosuppressant use) may limit applicability to more complex cases. Sixth, the 7‐day threshold was derived from a single high‐volume center and was not externally validated; centers with different capacity or transfer practices may require its recalibration. Seventh, our model applies specifically to patients requiring Neuro‐ICU care beyond 48 h, and its predictions should be interpreted within this intended population rather than extrapolated to all supratentorial tumor patients. Future studies should prospectively evaluate the proposed nomogram to ascertain its impact on hard endpoints such as Neuro‐ICU LOS and cost‐effectiveness. Exploring these inflammatory indices in other neurosurgical conditions (infratentorial tumors, aneurysms, stroke, etc.) represents another promising direction.

## 5 Conclusion

Early postoperative SIRI, PLR, and LMR independently predict long‐term Neuro‐ICU stay (≥ 7 days) in patients requiring prolonged care (> 48 h) after elective supratentorial brain tumor resection. An integrated early prediction model combining these inflammatory indices with three readily available clinical variables provides reliable risk stratification within the first 24 postoperative hours. The nomogram and web‐based calculator derived from this model may facilitate early identification of high‐risk patients and optimize resource allocation, pending validation in prospective, multi‐institutional cohorts.

## Author Contributions

Biwu Wu, Jin Hu, and Gang Wu contributed to the conception and design of the study. Haoyue Yuan and Yu Guo performed data collection. Biwu Wu carried out statistical analysis, data visualization, and figure preparation. Biwu Wu, Lichao Wei, Jin Hu, and Gang Wu contributed to data interpretation and critical discussion. Biwu Wu drafted the manuscript. All authors have made significant contributions to this study and have approved the final manuscript.

## Funding

This work was supported by National Science and Technology Major Projects for the Prevention and Treatment of Cancer, Cardiovascular and Cerebrovascular Diseases, Respiratory and Metabolic Diseases (2023ZD0505003); National Major Scientific Instruments and Equipments Development Project of National Natural Science Foundation of China (82427808).

## Disclosure

The authors have nothing to report.

## Ethics Statement

This study was approved by the Institutional Review Board of Huashan Hospital (March 31, 2025, no. 2025‐648) and registered at Chinese Clinical Trial Registry (no. ChiCTR2500104426).

## Conflicts of Interest

The authors declare no conflicts of interest.

## Supporting information


**Table S1:** Characteristics of short‐term and long‐term stay patients in the derivation cohort.
**Table S2:** Comparison of preoperative laboratory tests in the derivation cohort.
**Table S3:** Variance inflation factors for predictors in the constructed models.
**Table S4:** Discriminative performance of the five indices for long‐term stay.
**Table S5:** Comparison of predictive models and incremental predictive value for long‐term stay.
**Table S6:** Diagnostic performance of models and inflammatory indices at optimal cutoffs.
**Table S7:** Baseline characteristics and outcomes before and after propensity score matching.
**Table S7:** (Continued).
**Table S8:** Sensitivity analyses for the predictive value of SIRI.
**Table S9:** Model performance under an alternative temporal partition by calendar year.
**Table S10:** Sensitivity analyses of the integrated model with respect to airway management and perioperative factors (derivation cohort).
**Table S11:** Preoperative and early postoperative inflammatory indices in the derivation cohort.
**Table S12:** Extended model incorporating additional early clinical variables (derivation cohort).
**Table S13:** Comorbidity and functional status score and long‐term stay (derivation cohort).
**Figure S1:** Spearman correlation heatmap of candidate predictive features. The correlogram illustrates pairwise correlation coefficients among 41 candidate clinical, laboratory, and outcome variables. Positive and negative correlations are indicated by red and blue colors, respectively, with color intensity reflecting the correlation strength (ranging from −1 to 1).
**Figure S2:** Covariate balance before and after propensity score matching (PSM). The love plot illustrates absolute mean differences for baseline covariates between the compared groups. Following PSM, these differences were substantially reduced across all variables, indicating successful cohort balancing. PSM, propensity score matching; SBP, systolic blood pressure; Neuro‐ICU, neurosurgical intensive care unit; GCS, Glasgow Coma Scale; KPS, Karnofsky Performance Status; ETI, endotracheal intubation.

**Data S1:** cns71100‐sup‐0002‐DataS1.doc.

**Data S2:** cns71100‐sup‐0003‐DataS2.pdf.

## Data Availability

The data that support the findings of this study are available from the corresponding author upon reasonable request.
