# Supplementary material for: Early Postoperative Inflammatory Indices Predict Long‐Term Neuro‐ICU Stay in Patients Requiring Prolonged Care After Supratentorial Brain Tumor Resection: Development and Validation of an Integrated Model
Source: CNS Neurosci Ther. 2026 Aug 22;32(8):e71100. doi: 10.1002/cns.71100 (PMC13498967; doi:10.1002/cns.71100)
Supplement: Supplementary file 2 [file CNS-32-e71100-s002.docx]

**Supplementary Materials**

| **Table S1. Characteristics of short-term and long-term stay patients in the derivation cohort** | | | |
| --- | --- | --- | --- |
| **Clinical Variables** | **Short-term stay (n = 227)** | **Long-term stay** **(n = 205)** | ***P* value** |
| Age, years^†^ | 58.0 (46.5, 65.0) | 61.0 (49.0, 68.0) | 0.041 |
| Female^†^ | 125 (55.1%) | 92 (44.9%) | 0.044 |
| Hypertension | 100 (44.1%) | 106 (51.7%) | 0.135 |
| Diabetes^†^ | 48 (21.1%) | 64 (31.2%) | 0.023 |
| Preoperative KPS^†^ | 90.0 (80.0, 90.0) | 90.0 (70.0, 90.0) | 0.003 |
| Tumor type^†^ |  |  | 0.047 |
| High-grade glioma | 94 (41.4%) | 114 (55.6%) |  |
| Meningioma | 62 (27.3%) | 45 (22%) |  |
| Pituitary adenoma | 26 (11.5%) | 11 (5.4%) |  |
| Metastasis | 18 (7.9%) | 12 (5.9%) |  |
| Low-grade glioma | 14 (6.2%) | 13 (6.3%) |  |
| Other | 13 (5.7%) | 10 (4.9%) |  |
| Tumor site^†^ |  |  | 0.049 |
| Lobe | 141 (62.1%) | 130 (63.4%) |  |
| Skull base | 55 (24.2%) | 36 (17.6%) |  |
| Basal ganglia | 24 (10.6%) | 20 (9.8%) |  |
| Ventricle | 6 (2.6%) | 14 (6.8%) |  |
| Pineal region | 1 (0.4%) | 5 (2.4%) |  |
| Tumor diameter, cm | 4.9 (3.3, 6.0) | 5.0 (4.0, 6.0) | 0.171 |
| Tumor status |  |  | 0.258 |
| Initial onset | 204 (89.9%) | 176 (85.9%) |  |
| Recurrence | 23 (10.1%) | 29 (14.1%) |  |
| Surgical approach^†^ |  |  | 0.002 |
| Craniotomy | 170 (74.9%) | 180 (87.8%) |  |
| Endoscopic surgery | 34 (15%) | 13 (6.3%) |  |
| Keyhole surgery | 23 (10.1%) | 12 (5.9%) |  |
| Operative time, hours^†^ | 5.0 (4.0, 6.0) | 5.5 (4.0, 7.0) | 0.004 |
| Intraoperative blood loss, ml | 400.0 (300.0, 500.0) | 400.0 (300.0, 600.0) | 0.111 |
| Intraoperative RBC transfusion |  |  | 0.404 |
| ≤ 400 ml | 206 (90.7%) | 180 (87.8%) |  |
| ＞ 400 ml | 21 (9.3%) | 25 (12.2%) |  |
| Postoperative steroid use^†^ | 178 (78.4%) | 142 (69.3%) | 0.040 |
| Neuro-ICU admission GCS score^†^ | 4.0 (3.0, 12.0) | 3.0 (3.0, 7.0) | 0.010 |
| Postoperative SBP, mmHg | 137.8 ± 18.3 | 139.4 ± 20.0 | 0.366 |
| Postoperative DBP, mmHg | 81.6 ± 11.6 | 83.0 ± 12.4 | 0.195 |
| Postoperative SpO_2_, %^†^ | 99.0 (99.0, 100.0) | 99.0 (98.0, 100.0) | 0.026 |
| Postoperative heart rate | 88.0 (77.0, 100.0) | 88.0 (77.0, 99.0) | 0.916 |
| Postoperative body temperature, °C | 36.7 (36.5, 37.0) | 36.7 (36.5, 36.9) | 0.085 |
| Time to blood sampling, hours | 11.0 (8.0, 13.5) | 11.0 (8.0, 13.5) | 0.810 |

| **Table S1. (Continued)** | | | | |
| --- | --- | --- | --- | --- |
| Retained ETI at blood sampling^†^ | 108 (47.6%) | 161 (78.5%) | <0.001 | |
| White blood cells, ×10^9^/L | 13.3 (11.1, 16.4) | 14.9 (12.3, 17.8) | <0.001 | |
| Neutrophil count, ×10^9^/L | 12.2 (9.7, 14.4) | 13.6 (11.2, 16.5) | <0.001 | |
| Lymphocyte count, ×10^9^/L | 0.7 (0.5, 1.0) | 0.6 (0.4, 0.8) | <0.001 | |
| Monocyte count, ×10^9^/L | 0.4 (0.3, 0.6) | 0.6 (0.4, 0.9) | <0.001 | |
| Red blood cells, ×10^12^/L^†^ | 4.0 ± 0.5 | 4.1 ± 0.5 | 0.028 | |
| Hemoglobin, g/L^†^ | 119.0 (109.0, 129.5) | 121.0 (113.0, 135.0) | 0.014 | |
| Platelet, ×10^9^/L | 182.0 (137.0, 217.5) | 177.0 (141.0, 213.0) | 0.548 |  |
| Alanine aminotransferase, U/L | 19.0 (14.0, 27.0) | 20.0 (15.0, 31.0) | 0.303 |  |
| Creatinine, μmol/L | 63.0 (51.5, 76.0) | 62.0 (54.0, 72.0) | 0.740 |  |
| Systemic inflammation response index^†^ | 6.9 (4.5, 10.1) | 13.3 (9.6, 21.1) | <0.001 |  |
| Neutrophil-to-lymphocyte ratio^†^ | 17.8 (11.8, 25.6) | 25.0 (17.4, 35.4) | <0.001 |  |
| Platelet-to-lymphocyte ratio^†^ | 264.3 (172.2, 351.9) | 314.5 (227.0, 475.0) | <0.001 |  |
| Lymphocyte-to-Monocyte Ratio^†^ | 1.8 (1.2, 2.5) | 1.0 (0.7, 1.4) | <0.001 |  |
| Systemic Immune-Inflammation Index^†^ | 3001.5 (1972.1, 4501.4) | 4446.9 (2865.0, 6607.0) | <0.001 |  |
| Prothrombin time, s | 11.2 (10.8, 11.7) | 11.3 (10.9, 11.8) | 0.060 |  |
| Activated partial thromboplastin time, s | 26.8 (25.2, 28.2) | 26.9 (25.4, 28.2) | 0.609 |  |
| Data are presented as n (%), mean ± SD, or median (IQR). KPS, Karnofsky Performance Status; RBC, red blood cells; Neuro-ICU, neurosurgical intensive care unit; GCS, Glasgow Coma Scale; SBP, systolic blood pressure; DBP, diastolic blood pressure; SpO_2_, peripheral oxygen saturation; ETI, endotracheal intubation.  † Variables included in the LASSO candidate pool; the total white blood cell count and the individual leukocyte subset counts were not entered because they are components of the composite indices. Tumor type and tumor site were entered as binary indicators: malignant (high-grade glioma, metastasis) versus non-malignant (meningioma, pituitary adenoma, low-grade glioma, other), and deep (skull base, basal ganglia, ventricle, pineal region) versus lobar. | | | |  |

| **Table S2. Comparison of preoperative laboratory tests in the derivation cohort** | | | |
| --- | --- | --- | --- |
| **Variables** | **S****hort-term stay (n = 227)** | **Long-term stay (n = 205)** | ***P* value** |
| White blood cells, ×10^9^/L | 6.3 (4.9, 7.6) | 6.2 (5.0, 7.8) | 0.756 |
| Neutrophil count, ×10^9^/L | 3.8 (2.8, 5.0) | 3.9 (3.0, 5.4) | 0.551 |
| Lymphocyte count, ×10^9^/L | 1.7 (1.4, 2.2) | 1.6 (1.3, 2.0) | 0.162 |
| Monocyte count, ×10^9^/L | 0.4 (0.3, 0.5) | 0.4 (0.3, 0.5) | 0.150 |
| Red blood cells, ×10^12^/L | 4.4 (4.1, 4.7) | 4.4 (4.1, 4.8) | 0.668 |
| Hemoglobin, g/L | 131.0 (123.0, 140.0) | 131.0 (123.0, 142.0) | 0.528 |
| Platelet, ×10^9^/L | 198.0 (157.5, 242.0) | 189.0 (163.0, 226.0) | 0.523 |
| Alanine aminotransferase, U/L | 17.0 (11.0, 28.5) | 16.0 (12.0, 26.0) | 0.769 |
| Creatinine, μmol/L | 69.0 (57.5, 81.0) | 69.0 (59.0, 81.0) | 0.764 |
| Systemic inflammation response index | 0.8 (0.5, 1.4) | 0.9 (0.6, 1.5) | 0.083 |
| Neutrophil-to-lymphocyte ratio | 2.2 (1.7, 3.2) | 2.4 (1.7, 3.5) | 0.085 |
| Platelet-to-lymphocyte ratio | 110.4 (95.3, 146.9) | 125.5 (93.0, 164.4) | 0.075 |
| Lymphocyte-to-Monocyte Ratio | 4.7 (3.4, 6.2) | 4.3 (3.3, 5.3) | 0.019 |
| Systemic Immune-Inflammation Index | 434.4 (294.0, 671.5) | 487.2 (319.2, 730.7) | 0.175 |
| Prothrombin time, s | 11.2 (10.8, 11.7) | 11.3 (10.9, 11.8) | 0.118 |
| Activated partial thromboplastin time, s | 27.0 (25.4, 28.2) | 26.8 (25.1, 28.2) | 0.605 |
| Data are presented as median (IQR). | | | |

| **Table S3. Variance inflation factors for predictors in the constructed models** | | | |
| --- | --- | --- | --- |
| **Variables** | **Variance inflation factors** | | |
|  | **Model 1** | **Model 2** | **Model 3** |
| Preoperative Karnofsky Performance Status | 1.00 | 1.02 | 1.05 |
| Postoperative steroid use | 1.08 | 1.15 | 1.17 |
| Retained endotracheal intubation at blood sampling | 1.08 | 1.15 | 1.29 |
| Systemic inflammation response index | — | 1.97 | 1.97 |
| Platelet-to-lymphocyte ratio | — | 1.06 | 1.10 |
| Lymphocyte-to-monocyte ratio | — | 1.85 | 1.90 |
| Prolonged endotracheal intubation (at 48h) | — | — | 1.43 |
| Pulmonary infection (≤ 48h) | — | — | 1.21 |
| Postoperative hemorrhage (≤ 48h) | — | — | 1.25 |
| Unplanned reoperation (≤ 48h) | — | — | 1.23 |
| "—", variable not included in the model. | | | |

| **Table S4. Discriminative performance of the five indices for long-term stay** | | |
| --- | --- | --- |
|  | **Derivation cohort (n = 432)** | **Validation cohort (n = 146)** |
| **Discriminative performance** | **AUC (95% CI)** | **AUC (95% CI)** |
| SIRI | 0.779 (0.735–0.823) | 0.782 (0.708–0.856) |
| LMR | 0.773 (0.729–0.817) | 0.781 (0.707–0.854) |
| NLR | 0.686 (0.636–0.735) | 0.662 (0.573–0.750) |
| SII | 0.670 (0.620–0.721) | 0.654 (0.565–0.743) |
| PLR | 0.621 (0.568–0.674) | 0.618 (0.527–0.709) |
| **Pairwise comparison** | ***P* value** | ***P* value** |
| SIRI vs. LMR | 0.565 | 0.941 |
| SIRI vs. NLR | < 0.001 | 0.013 |
| SIRI vs. SII | < 0.001 | 0.007 |
| SIRI vs. PLR | < 0.001 | 0.004 |
| LMR vs. NLR | 0.001 | 0.015 |
| LMR vs. SII | < 0.001 | 0.014 |
| LMR vs. PLR | < 0.001 | 0.003 |
| NLR vs. SII | 0.276 | 0.812 |
| NLR vs. PLR | < 0.001 | 0.196 |
| SII vs. PLR | 0.001 | 0.189 |
| AUC, area under the receiver operating characteristic curve; CI, confidence interval; LMR, lymphocyte-to-monocyte ratio; NLR, neutrophil-to-lymphocyte ratio; PLR, platelet-to-lymphocyte ratio; SII, systemic immune-inflammation index; SIRI, systemic inflammation response index. AUCs were compared using DeLong's test. | | |

| **Table S5. Comparison of predictive models and incremental predictive value for long-term stay** | | | | | | | | |
| --- | --- | --- | --- | --- | --- | --- | --- | --- |
| **Predictive Models** | **Discrimination (Reference: Model 2)** | | |  | **Incremental value (Reference: Model 1)** | | | |
|  | **AUC (95% CI)** | **AIC** | ***P* value**^†^ |  | **IDI (95% CI)** | ***P* value**^‡^ | **cNRI (95% CI)** | ***P* value**^‡^ |
| Model 1 (Clinical baseline) | 0.715 (0.667–0.763) | 542.10 | < 0.001 |  | / | / | / | / |
| Model 1 + PLR | 0.739 (0.692–0.785) | 523.16 | < 0.001 |  | 0.039 (0.019–0.058) | < 0.001 | 0.372 (0.189–0.556) | < 0.001 |
| Model 1 + SII | 0.764 (0.720–0.808) | 504.16 | < 0.001 |  | 0.074 (0.048–0.100) | < 0.001 | 0.472 (0.291–0.653) | < 0.001 |
| Model 1 + NLR | 0.769 (0.725–0.812) | 498.06 | < 0.001 |  | 0.085 (0.058–0.113) | < 0.001 | 0.526 (0.344–0.707) | < 0.001 |
| Model 1 + LMR | 0.819 (0.780–0.858) | 459.70 | 0.003 |  | 0.158 (0.124–0.192) | < 0.001 | 0.821 (0.652–0.990) | < 0.001 |
| Model 1 + SIRI | 0.836 (0.799–0.873) | 442.34 | 0.158 |  | 0.192 (0.154–0.230) | < 0.001 | 0.822 (0.653–0.991) | < 0.001 |
| Model 2 (Integrated) | **0.845 (0.809–0.881)** | **434.13** | Ref |  | **0.212 (0.173–0.251)** | < 0.001 | **0.981 (0.817–1.145)** | < 0.001 |
| Model 3 (+Complications) | 0.904 (0.877–0.931) | 360.86 | < 0.001 |  | — | — | — | — |
| AUC, area under receiver operating characteristic; CI, confidence interval; AIC, Akaike information criterion; IDI, integrated discrimination improvement; cNRI, continuous net reclassification improvement; PLR, platelet-to-lymphocyte ratio; SII, systemic immune-inflammation index; NLR, neutrophil-to-lymphocyte ratio; LMR, lymphocyte-to-monocyte ratio; SIRI, systemic inflammation response index.  Model 1: Preoperative KPS, postoperative steroid use, and retained endotracheal intubation at blood sampling.  Model 2: Model 1 + SIRI + PLR + LMR.  Model 3: Model 2 + late postoperative complications.  † DeLong's test comparing each model's AUC against Model 2; ‡ IDI and cNRI calculated with Model 1 as the reference; “/” reference model; “—” not applicable. | | | | | | | | |

| **Table S6. Diagnostic performance of models and inflammatory indices at optimal cutoffs** | | | | | | | |
| --- | --- | --- | --- | --- | --- | --- | --- |
| **Predictors** | **AUC** | **Cutoff** | **Sensitivity** | **Specificity** | **PPV** | **NPV** | **Accuracy** |
| **Model 2 (Integrated model)** | **0.845** | **> 0.449** | **81.0%** | **73.1%** | **73.1%** | **81.0%** | **76.9%** |
| Model 1 (Clinical model) | 0.715 | > 0.428 | 81.5% | 51.5% | 60.3% | 75.5% | 65.7% |
| SIRI | 0.779 | > 9.26 | 78.5% | 71.4% | 71.2% | 78.6% | 74.8% |
| LMR | 0.773 | < 1.22 | 68.3% | 74.4% | 70.7% | 72.2% | 71.5% |
| NLR | 0.686 | > 23.26 | 59.0% | 68.7% | 63.0% | 65.0% | 64.1% |
| PLR | 0.621 | > 397.06 | 37.6% | 81.5% | 64.7% | 59.1% | 60.6% |
| SII | 0.670 | > 3391.08 | 66.3% | 61.2% | 60.7% | 66.8% | 63.7% |
| AUC, area under receiver operating characteristic; PPV, positive predictive value; NPV, negative predictive value.  The optimal cutoffs were determined by maximizing the Youden Index within the original derivation cohort.  Model 1: Preoperative KPS, postoperative steroid use, and retained endotracheal intubation at blood sampling.  Model 2: Model 1 integrated with SIRI, PLR, and LMR. | | | | | | | |

| **Table S7. Baseline characteristics and outcomes before and after propensity score matching** | | | | | | | | | | |
| --- | --- | --- | --- | --- | --- | --- | --- | --- | --- | --- |
| **Clinical Variables** | **Before PSM** | | | |  | **After PSM** | | | | |
|  | **SIRI < 9.26 (n = 205)** | **SIRI ≥ 9.26 (n = 227)** | ***P* value** | **SMD** |  | **SIRI < 9.26 (n = 118)** | **SIRI ≥ 9.26 (n = 118)** | ***P* value** | **SMD** |  |
| ***Age, years*** | 58.00 (47.00, 67.00) | 60.00 (48.50, 67.00) | 0.606 | 0.019 |  | 60.00 (50.00, 69.00) | 61.00 (49.00, 67.00) | 0.680 | 0.079 |  |
| ***Female*** | 115 (56.1%) | 102 (44.9%) | 0.026 | 0.225 |  | 63 (53.4%) | 62 (52.5%) | 1.000 | 0.017 |  |
| ***Hypertension*** | 82 (40.0%) | 124 (54.6%) | 0.003 | 0.296 |  | 58 (49.2%) | 65 (55.1%) | 0.434 | 0.119 |  |
| ***Diabetes*** | 50 (24.4%) | 62 (27.3%) | 0.560 | 0.067 |  | 34 (28.8%) | 31 (26.3%) | 0.771 | 0.057 |  |
| ***Preoperative KPS*** | 90.00 (80.00, 90.00) | 90.00 (80.00, 90.00) | 0.110 | 0.188 |  | 90.00 (80.00, 90.00) | 90.00 (80.00, 90.00) | 0.833 | 0.057 |  |
| ***Tumor type*** |  |  | 0.002 | 0.432 |  |  |  | 0.452 | 0.285 |  |
| High-grade gliom | 83 (40.5%) | 125 (55.1%) |  |  |  | 52 (44.1%) | 58 (49.2%) |  |  |  |
| Meningioma | 55 (26.8%) | 52 (22.9%) |  |  |  | 36 (30.5%) | 30 (25.4%) |  |  |  |
| Pituitary adenoma | 23 (11.2%) | 14 (6.2%) |  |  |  | 9 (7.6%) | 8 (6.8%) |  |  |  |
| Metastasis | 23 (11.2%) | 7 (3.1%) |  |  |  | 9 (7.6%) | 5 (4.2%) |  |  |  |
| Low-grade glioma | 11 (5.4%) | 16 (7.0%) |  |  |  | 8 (6.8%) | 7 (5.9%) |  |  |  |
| Other | 10 (4.9%) | 13 (5.7%) |  |  |  | 4 (3.4%) | 10 (8.5%) |  |  |  |
| ***Tumor site*** |  |  | 0.727 | 0.138 |  |  |  | 0.280 | 0.296 |  |
| Lobe | 126 (61.5%) | 145 (63.9%) |  |  |  | 80 (67.8%) | 75 (63.6%) |  |  |  |
| Skull base | 48 (23.4%) | 43 (18.9%) |  |  |  | 23 (19.5%) | 26 (22.0%) |  |  |  |
| Basal ganglia | 21 (10.2%) | 23 (10.1%) |  |  |  | 13 (11.0%) | 9 (7.6%) |  |  |  |
| Ventricle | 8 (3.9%) | 12 (5.3%) |  |  |  | 2 (1.7%) | 6 (5.1%) |  |  |  |
| Pineal region | 2 (1.0%) | 4 (1.8%) |  |  |  | 0 (0.0%) | 2 (1.7%) |  |  |  |
| ***Tumor diameter, cm*** | 4.70 (3.20, 6.00) | 5.10 (4.00, 6.15) | 0.011 | 0.258 |  | 5.00 (3.42, 6.27) | 5.05 (3.80, 5.77) | 0.848 | 0.034 |  |
| ***Surgical approach*** |  |  | 0.002 | 0.337 |  |  |  | 0.761 | 0.096 |  |
| Craniotomy | 152 (74.1%) | 198 (87.2%) |  |  |  | 102 (86.4%) | 98 (83.1%) |  |  |  |
| Endoscopic surgery | 31 (15.1%) | 16 (7.0%) |  |  |  | 10 (8.5%) | 12 (10.2%) |  |  |  |
| Keyhole surgery | 22 (10.7%) | 13 (5.7%) |  |  |  | 6 (5.1%) | 8 (6.8%) |  |  |  |
| ***Operative time, hours*** | 5.00 (4.00, 6.00) | 5.50 (4.50, 7.00) | 0.001 | 0.269 |  | 5.00 (4.00, 7.00) | 5.50 (4.00, 7.00) | 0.735 | 0.002 |  |
| ***Intraoperative blood loss, ml*** | 400.00 (200.00, 500.00) | 400.00 (300.00, 550.00) | 0.077 | 0.045 |  | 400.00 (300.00, 500.00) | 400.00 (300.00, 500.00) | 0.782 | 0.051 |  |
| ***Postoperative steroid use*** | 156 (76.1%) | 164 (72.2%) | 0.422 | 0.088 |  | 87 (73.7%) | 82 (69.5%) | 0.564 | 0.094 |  |

| **Table S7.** **(Continued)** | | | | | | | | | |  |
| --- | --- | --- | --- | --- | --- | --- | --- | --- | --- | --- |
| ***Neuro-ICU admission GCS score*** | 4.00 (3.00, 11.00) | 4.00 (3.00, 9.00) | 0.097 | 0.161 |  | 4.00 (3.00, 9.00) | 3.50 (3.00, 9.75) | 0.706 | 0.004 |  |
| ***Postoperative SBP, mmHg*** | 139.00 (123.00, 148.00) | 140.00 (128.00, 153.50) | 0.027 | 0.236 |  | 141.50 (127.50, 152.00) | 140.00 (129.00, 153.00) | 0.873 | 0.063 |  |
| ***Time to blood sampling, hours*** | 10.50 (7.50, 13.50) | 11.50 (8.00, 13.50) | 0.302 | 0.113 |  | 11.00 (8.00, 13.50) | 11.00 (8.00, 13.00) | 0.732 | 0.040 |  |
| ***Retained ETI at blood sampling*** | 113 (55.1%) | 156 (68.7%) | 0.005 | 0.283 |  | 75 (63.6%) | 78 (66.1%) | 0.785 | 0.053 |  |
| ***White blood cells, ×10^9^/L*** | 12.40 (9.91, 14.78) | 15.66 (12.95, 18.88) | <0.001 | 0.898 |  | 13.68 (11.73, 16.30) | 14.05 (12.09, 16.49) | 0.371 | 0.139 |  |
| ***Red blood cells, ×10^12^/L*** | 3.97 (3.57, 4.40) | 4.06 (3.76, 4.40) | 0.102 | 0.178 |  | 3.98 (3.62, 4.48) | 3.96 (3.72, 4.28) | 0.682 | 0.056 |  |
| ***Creatinine, μmol/L*** | 61.00 (51.00, 74.00) | 64.00 (55.00, 74.00) | 0.040 | 0.002 |  | 63.00 (51.00, 73.75) | 62.50 (55.00, 73.50) | 0.465 | 0.144 |  |
| Tumor status (Recurrence) | 20 (9.8%) | 32 (14.1%) | 0.216 | 0.134 |  | 9 (7.6%) | 15 (12.7%) | 0.282 | 0.169 |  |
| Intraoperative RBC transfusion | 25 (12.2%) | 21 (9.3%) | 0.404 | 0.095 |  | 20 (16.9%) | 15 (12.7%) | 0.464 | 0.119 |  |
| Postoperative DBP, mmHg | 82.00 (74.00, 89.00) | 83.00 (74.50, 91.00) | 0.286 | 0.128 |  | 83.50 (75.25, 91.75) | 82.50 (75.00, 91.75) | 0.801 | 0.004 |  |
| Postoperative SpO_2_, % | 99.00 (99.00, 100.00) | 99.00 (98.00, 100.00) | 0.155 | 0.103 |  | 99.00 (98.00, 100.00) | 99.00 (98.00, 100.00) | 0.406 | 0.132 |  |
| Postoperative heart rate | 88.00 (75.00, 99.00) | 89.00 (78.00, 100.00) | 0.419 | 0.079 |  | 90.00 (78.00, 100.00) | 90.50 (77.25, 100.00) | 0.999 | 0.001 |  |
| Postoperative body temperature, °C | 36.70 (36.50, 37.00) | 36.70 (36.50, 37.00) | 0.626 | 0.052 |  | 36.65 (36.50, 36.90) | 36.70 (36.50, 37.00) | 0.250 | 0.122 |  |
| Hemoglobin, g/L | 120.00 (108.00, 132.00) | 120.00 (113.00, 132.00) | 0.158 | 0.150 |  | 119.50 (109.50, 132.00) | 119.00 (111.00, 128.75) | 0.757 | 0.054 |  |
| Platelet, ×10^9^/L | 181.00 (136.00, 210.00) | 182.00 (141.50, 215.00) | 0.375 | 0.081 |  | 182.00 (142.00, 210.00) | 179.50 (137.50, 206.00) | 0.421 | 0.103 |  |
| Alanine aminotransferase, U/L | 19.00 (14.00, 32.00) | 20.00 (14.00, 29.00) | 0.844 | 0.015 |  | 20.00 (15.00, 34.00) | 19.00 (14.00, 27.75) | 0.101 | 0.067 |  |
| Prothrombin time, s | 11.30 (10.80, 11.80) | 11.30 (10.80, 11.70) | 0.433 | 0.040 |  | 11.30 (10.83, 11.70) | 11.30 (10.80, 11.70) | 0.956 | 0.078 |  |
| Activated partial thromboplastin time, s | 26.80 (25.20, 28.10) | 26.90 (25.25, 28.20) | 0.459 | 0.068 |  | 26.60 (25.13, 27.90) | 27.10 (25.70, 28.58) | 0.121 | 0.196 |  |
| Prolonged ETI (at 48h) | 34 (16.6%) | 74 (32.6%) | <0.001 | 0.378 |  | 23 (19.5%) | 37 (31.4%) | 0.052 | 0.275 |  |
| Seizures (≤ 48h) | 21 (10.2%) | 20 (8.8%) | 0.731 | 0.049 |  | 13 (11.0%) | 13 (11.0%) | 1.000 | <0.001 |  |
| Pulmonary infection (≤ 48h) | 55 (26.8%) | 154 (67.8%) | <0.001 | 0.901 |  | 36 (30.5%) | 70 (59.3%) | <0.001 | 0.605 |  |
| Postoperative hemorrhage (≤ 48h) | 33 (16.1%) | 94 (41.4%) | <0.001 | 0.582 |  | 21 (17.8%) | 47 (39.8%) | <0.001 | 0.502 |  |
| Unplanned reoperation (≤ 48h) | 14 (6.8%) | 24 (10.6%) | 0.230 | 0.133 |  | 8 (6.8%) | 16 (13.6%) | 0.132 | 0.226 |  |
| Long-term Neuro-ICU stay (≥ 7d) | 44 (21.5%) | 161 (70.9%) | <0.001 | 1.143 |  | 32 (27.1%) | 75 (63.6%) | <0.001 | 0.787 |  |
| Neuro-ICU length of stay, days | 5.00 (3.00, 6.00) | 7.00 (5.00, 11.00) | <0.001 | 0.695 |  | 5.00 (4.00, 7.00) | 7.00 (5.00, 11.00) | <0.001 | 0.537 |  |
| Data are presented as n (%) or median (IQR). PSM, propensity score matching; KPS, Karnofsky Performance Status; Neuro-ICU, neurosurgical intensive care unit; GCS, Glasgow Coma Scale; SBP, systolic blood pressure; ETI, endotracheal intubation; RBC, red blood cells; NA, not available; DBP, diastolic blood pressure; SpO_2_, peripheral oxygen saturation.  Note: PSM (1:1 nearest neighbor, caliper = 0.20) was performed. The 19 covariates (bold and italic) include: Pre-specified (Age, Gender, Diabetes, Preoperative KPS, Tumor type, Tumor site, Tumor diameter, Surgical approach, Operative time, Intraoperative blood loss, Postoperative steroid use, Neuro-ICU admission GCS score, Time to blood sampling, Retained ETI at blood sampling) and *P* < 0.1 (Hypertension, Postoperative SBP, White blood cells, Red blood cells, Creatinine). | | | | | | | | | | |

| **Table S8. Sensitivity analyses for the predictive value of SIRI** | | |
| --- | --- | --- |
| **Part A. Conditional logistic regression in the PSM cohort** | | |
| **Variables** | **OR (95% CI)** | ***P* value** |
| Continuous approach |  |  |
| SIRI (per 1-unit increase) | 1.24 (1.11–1.38) | < 0.001 |
| Categorical approach |  |  |
| SIRI < 9.26 (Low risk) | Reference |  |
| SIRI ≥ 9.26 (High risk) | 9.60 (3.82–24.11) | < 0.001 |
| **Part B. Model 2 performance across LTS thresholds** | | |
| **Outcome definition** | **Derivation cohort (AUC, 95% CI)** | **Validation cohort (AUC, 95% CI)** |
| LTS ≥ 4 days | 0.751 (0.688–0.814) | 0.752 (0.603–0.902) |
| LTS ≥ 5 days | 0.754 (0.707–0.802) | 0.737 (0.648–0.826) |
| LTS ≥ 6 days | 0.789 (0.747–0.832) | 0.768 (0.692–0.844) |
| **LTS ≥ 7 days (Primary)** | **0.845 (0.809–0.881)** | **0.816 (0.748–0.884)** |
| LTS ≥ 8 days | 0.809 (0.769–0.850) | 0.840 (0.773–0.907) |
| LTS ≥ 9 days | 0.777 (0.732–0.822) | 0.802 (0.724–0.880) |
| LTS ≥ 10 days | 0.765 (0.716–0.813) | 0.784 (0.700–0.868) |
| SIRI, systemic inflammation response index; PSM, propensity score matching; OR, odds ratio; CI, confidence interval; LTS, long-term stay; AUC, area under receiver operating characteristic.  Model 2: Preoperative KPS, postoperative steroid use, retained endotracheal intubation at blood sampling, SIRI, PLR, and LMR. | | |

| **Table S9. Model performance under an alternative temporal partition by calendar year** | | |
| --- | --- | --- |
|  | **Derivation cohort (2023)** | **Validation cohort (2024)** |
| **Cohort characteristics** |  |  |
| Patients, n | 363 | 215 |
| Long-term stay, n (%) | 167 (46.0) | 110 (51.2) |
| Events per variable | 8.8 | — |
| **Model discrimination, AUC (95% CI)** |  |  |
| Model 1 | 0.715 (0.663–0.768) | 0.661 (0.589–0.733) |
| Model 2 | 0.833 (0.793–0.873) | 0.835 (0.783–0.888) |
| Model 3 | 0.896 (0.866–0.927) | 0.885 (0.842–0.929) |
| **Calibration and performance of Model 2** |  |  |
| Brier score | 0.167 | 0.165 |
| Calibration slope | 0.938 | 1.095 |
| Hosmer-Lemeshow test, p value | 0.198 | 0.032 |
| Optimism-corrected AUC | 0.823 | — |
| IDI versus Model 1 (95% CI) | 0.189 (0.147–0.230) | — |
| cNRI versus Model 1 (95% CI) | 0.928 (0.746–1.111) | — |
| Sensitivity / specificity, % | 79.0 / 75.0 | — |
| **Multivariable analysis of Model 2 in the derivation cohort** | | |
| **Variable** | **OR (95% CI)** | ***P* value** |
| Preoperative KPS | 0.98 (0.96–1.00) | 0.029 |
| Postoperative steroid use | 0.37 (0.20–0.69) | 0.002 |
| Retained ETI at blood sampling | 5.89 (3.32–10.45) | < 0.001 |
| SIRI | 1.09 (1.05–1.15) | < 0.001 |
| PLR (per 100-unit increase) | 1.19 (1.03–1.39) | 0.021 |
| LMR | 0.72 (0.50–1.05) | 0.086 |
| AUC, area under the receiver operating characteristic curve; CI, confidence interval; cNRI, continuous net reclassification improvement; ETI, endotracheal intubation; IDI, integrated discrimination improvement; KPS, Karnofsky Performance Status; LMR, lymphocyte-to-monocyte ratio; OR, odds ratio; PLR, platelet-to-lymphocyte ratio; SIRI, systemic inflammation response index; "—", not applicable.  The incidence of long-term stay did not differ between the two periods (*p* = 0.266).  Discrimination of Model 2 did not differ between cohorts (DeLong's test, *p* = 0.941).  Calibration in the derivation cohort was assessed with bootstrap correction (1000 resamples). | | |

| **Table S10. Sensitivity analyses of the integrated model with respect to airway management and perioperative factors (derivation cohort)** | | | | | | | |
| --- | --- | --- | --- | --- | --- | --- | --- |
| **Airway variable used in the model** | | **Derivation AUC (95% CI)** | | **Validation AUC (95% CI)** | | | **AIC** |
| Retained ETI at blood sampling (Model 2) | | 0.845 (0.809–0.881) | | 0.816 (0.748–0.884) | | | 434.13 |
| Retained ETI at 24 h | | 0.871 (0.838–0.905) | | 0.870 (0.813–0.928) | | | 399.09 |
| Retained ETI at 48 h | | 0.874 (0.841–0.907) | | 0.852 (0.791–0.913) | | | 397.31 |
| None (airway variable removed) | | 0.803 (0.761–0.844) | | 0.802 (0.732–0.872) | | | 477.94 |
| **Inflammatory indices under each specification, OR (95% CI)** | | | | | | | |
| **Airway variable used in the model** | | **SIRI** | | **PLR (per 100 units)** | | | **LMR** |
| Retained ETI at blood sampling (Model 2) | | 1.11 (1.06–1.16) | | 1.19 (1.03–1.37) | | | 0.68 (0.45–0.96) |
| Retained ETI at 24 h | | 1.11 (1.06–1.17) | | 1.20 (1.03–1.39) | | | 0.71 (0.49–1.03) |
| Retained ETI at 48 h | | 1.09 (1.04–1.14) | | 1.26 (1.07–1.47) | | | 0.60 (0.40–0.88) |
| None (airway variable removed) | | 1.08 (1.04–1.13) | | 1.21 (1.05–1.38) | | | 0.62 (0.42–0.91) |
| **Model 2 with further adjustment for a perioperative factor** | | | | | | | |
| **Factor added to Model 2** | **OR of the added factor (95% CI)** | | ***P* value** | | **Derivation AUC** | **SIRI, OR (95% CI)** | |
| Time to blood sampling | 1.01 (0.94–1.08) | | 0.871 | | 0.844 | 1.11 (1.06–1.16) | |
| Operative time | 1.08 (0.97–1.20) | | 0.147 | | 0.845 | 1.11 (1.06–1.16) | |
| Surgical approach | 0.74 (0.47–1.16) | | 0.194 | | 0.844 | 1.11 (1.06–1.16) | |
| AIC, Akaike information criterion; AUC, area under the receiver operating characteristic curve; CI, confidence interval; ETI, endotracheal intubation; LMR, lymphocyte-to-monocyte ratio; OR, odds ratio; PLR, platelet-to-lymphocyte ratio; SIRI, systemic inflammation response index.  Model 2: preoperative KPS, postoperative steroid use, retained ETI at blood sampling, SIRI, PLR, and LMR.  Removing the airway variable reduced discrimination relative to Model 2 (DeLong's test, *p* = 0.001).  *P* values for SIRI were < 0.001 in every specification; those for PLR ranged from 0.004 to 0.021 and those for LMR from 0.010 to 0.068. | | | | | | | |

| **Table S11. Preoperative and early postoperative inflammatory indices in the derivation cohort** | | | | | | | |
| --- | --- | --- | --- | --- | --- | --- | --- |
| **Index** | **Preoperative**  **median (IQR)** | **Early postoperative**  **median (IQR)** | | **Preoperative AUC (95% CI)** | | **Early postoperative AUC (95% CI)** | ***P* value** |
| SIRI | 0.9 (0.6, 1.5) | 9.6 (5.8, 16.5) | | 0.548 (0.494–0.602) | | 0.779 (0.735–0.823) | < 0.001 |
| NLR | 2.3 (1.7, 3.4) | 21.2 (14.0, 29.8) | | 0.548 (0.494–0.602) | | 0.686 (0.636–0.735) | < 0.001 |
| PLR | 117.4 (94.1, 157.6) | 293.2 (197.2, 421.2) | | 0.550 (0.495–0.605) | | 0.621 (0.568–0.674) | 0.012 |
| LMR | 4.5 (3.3, 5.8) | 1.3 (0.8, 2.0) | | 0.565 (0.511–0.619) | | 0.773 (0.729–0.817) | < 0.001 |
| SII | 457.3 (304.0, 725.6) | 3502.5 (2353.9, 5539.2) | | 0.538 (0.483–0.592) | | 0.670 (0.620–0.721) | < 0.001 |
| **Model 2 with additional adjustment for the corresponding preoperative values** | | | | | | | |
| **Variable** | | | **OR (95% CI)** | | ***P* value** | | |
| **Early postoperative indices** | | |  | |  | | |
| SIRI | | | 1.11 (1.06–1.17) | | < 0.001 | | |
| PLR (per 100-unit increase) | | | 1.24 (1.05–1.46) | | 0.010 | | |
| LMR | | | 0.71 (0.49–1.03) | | 0.071 | | |
| **Preoperative indices** | | |  | |  | | |
| SIRI | | | 0.80 (0.52–1.25) | | 0.329 | | |
| NLR | | | 1.14 (0.90–1.46) | | 0.279 | | |
| PLR (per 100-unit increase) | | | 0.74 (0.36–1.54) | | 0.419 | | |
| LMR | | | 0.95 (0.80–1.12) | | 0.509 | | |
| SII (per 1000-unit increase) | | | 0.74 (0.21–2.61) | | 0.640 | | |
| AUC, area under the receiver operating characteristic curve; CI, confidence interval; IQR, interquartile range; LMR, lymphocyte-to-monocyte ratio; NLR, neutrophil-to-lymphocyte ratio; OR, odds ratio; PLR, platelet-to-lymphocyte ratio; SII, systemic immune-inflammation index; SIRI, systemic inflammation response index.  All indices changed significantly from before to after surgery (Wilcoxon signed-rank test, all *p* < 0.001).  *P* values in the upper panel refer to the comparison of preoperative and early postoperative AUCs (DeLong's test).  The lower panel shows Model 2 refitted with the preoperative value of each index added, and therefore indicates whether the early postoperative values remain predictive once individual preoperative levels are taken into account.  The model also includes preoperative KPS, postoperative steroid use, and retained ETI at blood sampling. | | | | | | | |

| **Table S12. Extended model incorporating additional early clinical variables (derivation cohort)** | | | | | | |
| --- | --- | --- | --- | --- | --- | --- |
| **Variable** | | **Univariable OR (95% CI)** | ***P* value** | | **Adjusted OR (95% CI)** | ***P* value** |
| Neuro-ICU admission GCS score | | 0.94 (0.90–0.98) | 0.006 | | 1.04 (0.96–1.12) | 0.333 |
| Deep tumor location | | 0.95 (0.64–1.40) | 0.780 | | 1.09 (0.65–1.81) | 0.746 |
| Ventricular or pineal location | | 3.21 (1.32–7.81) | 0.010 | | 3.25 (1.03–10.25) | 0.044 |
| Postoperative SpO_2_ | | 0.87 (0.75–1.00) | 0.056 | | 0.85 (0.70–1.02) | 0.079 |
| Postoperative systolic blood pressure | | 1.00 (0.99–1.01) | 0.366 | | 1.00 (0.99–1.01) | 0.931 |
| Age | | 1.01 (1.00–1.02) | 0.171 | | 1.01 (0.99–1.03) | 0.307 |
| **Discrimination** | | | | | | |
| **Model** | **Derivation AUC (95% CI)** | | | **Validation AUC (95% CI)** | | |
| Model 2 | 0.845 (0.809–0.881) | | | 0.816 (0.748–0.884) | | |
| Extended model | 0.849 (0.813–0.884) | | | 0.809 (0.740–0.879) | | |
| AUC, area under the receiver operating characteristic curve; CI, confidence interval; GCS, Glasgow Coma Scale; Neuro-ICU, neurosurgical intensive care unit; OR, odds ratio; SpO_2_, peripheral oxygen saturation.  Deep tumor location denotes a tumor of the skull base, basal ganglia, ventricle, or pineal region, as opposed to a lobar tumor.  The extended model comprises the six variables of Model 2 plus the five variables listed, and did not differ from Model 2 in discrimination (DeLong's test, p = 0.227).  Ventricular or pineal location was assessed separately in an otherwise identical model in which it replaced deep tumor location, the two being nested. Tumors at these sites were present in 26 patients in the derivation cohort and 5 in the validation cohort, and this variable was therefore not carried forward into the primary model. | | | | | | |

| **Table S13. Comorbidity and functional status score and long-term stay (derivation cohort)** | | | | | | | |
| --- | --- | --- | --- | --- | --- | --- | --- |
| **Index score** | | **Patients, n** | | | | **Long-term stay, n (%)** | |
| 0 | | 169 | | | | 66 (39.1) | |
| 1 | | 183 | | | | 90 (49.2) | |
| 2 | | 71 | | | | 44 (62.0) | |
| 3 | | 9 | | | | 5 (55.6) | |
| **Association with long-term stay** | | | | | | | |
| **Index definition** | **Univariable OR (95% CI)** | | | ***P* value** | **Adjusted OR (95% CI)** | | ***P* value** |
| Hypertension + diabetes | 1.42 (1.08–1.85) | | | 0.011 | 1.08 (0.78–1.51) | | 0.629 |
| Hypertension + diabetes + KPS < 70 | 1.50 (1.17–1.93) | | | 0.002 | 1.08 (0.78–1.49) | | 0.656 |
| **Discrimination** | | | | | | | |
| **Model** | | | **Derivation AUC (95% CI)** | | | | |
| Model 2 | | | 0.845 (0.809–0.881) | | | | |
| Model 2 plus the index | | | 0.844 (0.808–0.880) | | | | |
| CI, confidence interval; KPS, Karnofsky Performance Status; OR, odds ratio.  One point was assigned for each of hypertension, diabetes, and preoperative KPS below 70; the upper and lower panels refer to this three-component definition. Chronic obstructive pulmonary disease and congestive heart failure were not routinely recorded and could not be included.  Long-term stay was more frequent at higher index scores (chi-square test for trend, *p* = 0.011).  Adjusted estimates derive from models comprising the six variables of Model 2 plus the index. | | | | | | | |

**Figure S1. Spearman correlation heatmap of candidate predictive features.** The correlogram illustrates pairwise correlation coefficients among 41 candidate clinical, laboratory, and outcome variables. Positive and negative correlations are indicated by red and blue colors, respectively, with color intensity reflecting the correlation strength (ranging from -1 to 1).

**Figure S2. Covariate balance before and after propensity score matching (PSM).** The love plot illustrates absolute mean differences for baseline covariates between the compared groups. Following PSM, these differences were substantially reduced across all variables, indicating successful cohort balancing. PSM, propensity score matching; SBP, systolic blood pressure; Neuro-ICU, neurosurgical intensive care unit; GCS, Glasgow Coma Scale; KPS, Karnofsky Performance Status; ETI, endotracheal intubation.
